# The effect of the oral administration of lactic-acid probiotic bacteria on the vaginal microflora of bitches

**DOI:** 10.2478/jvetres-2025-0041

**Published:** 2025-08-07

**Authors:** Piotr Andrzej Socha, Sławomir Zduńczyk

**Affiliations:** Department of Animal Reproduction with Clinic, Faculty of Veterinary Medicine, University of Warmia and Mazury in Olsztyn, 10-719 Olsztyn, Poland

**Keywords:** dog, protective antimicrobial

## Abstract

**Introduction:**

In recent years, probiotics have been increasingly used in companion animals. However, there are few studies on their effect on canine vaginal flora. The objective of this study was to evaluate the effect of orally administrated multi-strain lactic-acid probiotic bacteria on the vaginal flora of healthy bitches.

**Material and Methods:**

A total of 38 Labrador retriever bitches were given one sachet of multi-strain probiotic with food once a day for nine weeks. Samples for microbiological examination were taken twice (on day 0 and day 63) from the cranial vagina and cultured for aerobic and anaerobic bacteria.

**Results:**

Bacterial growth was found in 84.2% of the samples on day 0 and in 94.7% of the samples on day 63. Mixed cultures were found in 63.1% of bitches with positive bacterial tests on day 0 and in 73.6% on day 63, and contained a mean 1.8 bacterial strains. An increase in the prevalence of *E. coli*, Gram-negative rods other than *E. coli, Staphylococcus* spp. and *Streptococcus* spp./ *Enterococcus* spp. was noted after nine weeks (P < 0.05). After the oral administration of the lactic-acid probiotic bacteria, potentially pathogenic *Clostridium perfringens, Canicola haemoglobinophilus* and *Pseudomonas aeruginosa* were not isolated. The prevalence of *Streptococci* other than *Streptococcus canis* was significantly higher after the administration of the probiotic (26.3% *vs* 5.3%, P < 0.05), and *Enterococcus* spp. were isolated in the samples of 28.9% of bitches. There was no increase in the prevalence of *Lactobacillus* spp.

**Conclusion:**

The results indicate a beneficial effect of oral lactic-acid probiotic bacteria on the composition of vaginal flora in bitches; however, further research is needed.

## Introduction

The vagina of bitches contains physiological microflora consisting of aerobic and anaerobic microorganisms which are mostly opportunistic pathogens (11, 14, 20, 21, 24, 35, 41). The vaginal microflora are usually mixed, composed of bacteria such as *E. coli*, beta-haemolytic and non beta-haemolytic *Streptococci* spp., coagulase-positive *Staphylococci* and those in the *Pasteurellales* order (24). Its composition varies depending on various factors, such as reproductive status (27, 33, 37), stage of oestrous cycle (4, 11, 13, 21, 41), breed (4) and age (18, 33).

Growth of bacteria resulted in 50–90% of cultured vaginal smears (11, 14, 24, 33). Not only experimentally but also in practice, the efficient culture-based methods are still widely used; currently, no other method exists to replace them completely (24). In recent years, however, culture-independent studies using next-generation sequencing have begun to be used to investigate the vaginal microbiome in bitches. *Proteobacteria, Bacteroidetes* and *Firmicutes* were the most abundant phyla found in the vagina in culture-based and culture-independent investigations (13, 24, 27, 38).

The importance of the vaginal bacterial flora in fertility disorders of bitches is not fully elucidated. On one hand, changes in the vaginal flora were often associated with reproductive tract diseases (7). *Escherichia coli*, beta-haemolytic *Streptococci, Staphylococcus intermedius* and *Pasteurella multocida* were the species most often isolated from bitches with pyometra and from those with dead puppies (3, 14, 34, 42). On the other hand, numerous studies showed that bacterial species isolated from bitches with reproductive disorders did not generally differ from those found in healthy bitches (11, 20, 24, 44). Samples from bitches with vaginitis tended to have fewer species but a higher number of bacteria than samples from healthy ones (3, 20, 36, 44).

There is a consensus that a positive vaginal culture alone without clinical symptoms should not be sufficient indication for antibiotic treatment (11, 14, 20, 24). Non-indicated antibiotic treatment promotes the development of antimicrobial bacterial resistance and disturbs the sensitive balance of the vaginal microbial environment (31, 39). In the vaginal microbiome, several strains of bacteria compete with each other for nutrients. Vaginal presence of *Streptococcus* spp. was negatively associated with the development of genital infections (11, 14, 20). Therefore, *Streptococcus* spp. may have a protective competitive role against more dangerous pathogens affecting fertility of the bitch.

Studies in humans suggest that modulation of the composition of the vaginal bacterial flora and inhibition of overgrowth of pathogenic bacteria using probiotics appears to be a promising method of preventing genital infections (6, 30). The use of probiotics can increase beneficial bacteria, reduce the number of harmful bacteria and further maintain the stability of the human vaginal flora environment (26). Probiotics are defined as ‘living microorganisms, which when administered in adequate amounts confer health benefits on the host’ (9). As is known when they are administered orally, probiotics benefit host health by influencing the composition and function of the gut microbiome. They compete with pathogenic microorganisms for nutrients and adhesion areas and produce antimicrobial substances and metabolic compounds that suppress the growth of other microorganisms. Moreover, probiotics can modulate the intestinal immunity (23, 25, 43). Commonly used probiotics in dietary supplements are lactic acid-producing bacteria such as *Lactobacillus* spp., *Bifidobacterium* spp., and *Enterococcus* spp. (17).

In recent years, probiotics have been increasingly used in companion animals (23, 32, 43). Probiotic supplementations have been successful in the prevention and treatment of acute gastroenteritis, treatment of irritable bowel disease, and the prevention of allergy in companion animals (2, 15, 43). However, there are few studies on the effect of probiotics on vaginal flora in bitches. *In vitro* studies showed that probiotic lactic acid bacteria strains inhibited the growth of canine genital pathogens (7, 10). There is only one study on the effect of oral probiotic on the vaginal microbiota in spayed female dogs (19). The study showed that oral probiotic supplement for a two-or four-week period did not increase the prevalence of lactic-acid bacteria in the vagina. However, the other vaginal bacteria were not examined.

The aim of this study was to evaluate the effect of the administration of an oral lactic-acid probiotic bacteria for nine weeks on the vaginal flora of healthy bitches.

## Material and Methods

### Animals

A total of 38 Labrador retriever bitches from five kennels were enrolled in this study. The owners were informed about the purpose of the study and gave their written consent. The bitches were aged between 2 and 6 years and were clinically healthy. They were fed commercial dry food of various brands. Animals undergoing any previously commenced treatment were excluded. All bitches were in the anoestrous phase as determined by the interview (which established that their last parturition or heat had been two or three months beforehand) and by cytological examination of vaginal smears.

### Administration of the probiotic

The bitches were given 21g of an oral multi-strain lactic-acid probiotic (PetBIOM; Owlie, Stawiguda, Poland) with food once daily for nine weeks. The probiotic PetBIOM contains in total 2 × 10^11^ colony-forming units/kg of *Lactobacillus plantarum* AMT4 strain and AMT14 strain and *Bifidobacterium animalis* AMT30 strain, as well as products produced by patented strains of bacteria with probiotic properties.

### Sample collection

Samples for cytological and microbiological examination were taken from the cranial vagina using a sterile swab and a sterile vaginal specula for bitches (Eickemeyer, Tuttlingen, Germany). The microbiological samples were delivered to the laboratory in a refrigerated box within 4 h of collection.

### Cytological and microbiological examination

The cytological smears were prepared and examined according to a standard procedure as described previously (5). Microbiological examination was performed at the Department of Microbiology and Clinical Immunology, Faculty of Veterinary Medicine, University of Warmia and Mazury in Olsztyn, Poland. The swabs were pre-incubated in non-selective tryptic soy broth (Oxoid, Basingstoke, UK) at 37°C for 24 h under aerobic conditions. The samples were then inoculated onto four agars: Columbia supplemented with 5% defibrinated sheep blood, MacConkey, Edwards and Chapman (all from Oxoid). Bacteria were cultured under aerobic conditions at 37°C for 48 h. The grown isolates were subjected to microbiological analysis. The morphology of bacterial colonies was assessed, Gram staining was undertaken and selected biochemical tests (catalase, coagulase and oxidase tests; and analytical profile index (API) 20 for Enterobacteriaceae (20E) and API 20 for non-enteric bacteria (20NE) tests; bioMérieux, Marcy-l’Étoile, France), the Christie–Atkins–Munch-Peterson reaction and selected latex agglutination tests (PathoDxtra Strep grouping kit and Staphytect Plus; Oxoid) were conducted. The medium for the anaerobic bacteria culture was CHROMagar *Clostridium perfringens* medium (CHROMAgar; Saint-Denis, France). Anaerobic cultures were incubated at 37°C in an anaerobic chamber with the Gaspak system (Oxoid). Subsequently, grown colonies were identified by Gram staining and the API 20 for anaerobic bacteria (20A) test.

### Statistical analysis

The prevalence of bacterial species was analysed using Fisher’s exact test in GraphPad Prism, v. 10.00 (GraphPad Software, San Diego, CA, USA). The level of significance was set at P-value < 0.05.

## Results

**Fig. 1.**
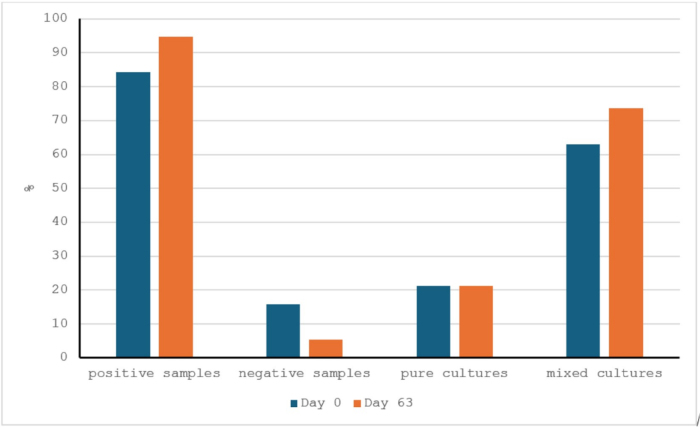
Isolation of bacteria from vaginal swab samples from Labrador retriever bitches (n = 38) before (day 0) and after (day 63) the oral administration of lactic-acid probiotic bacteria. The percentages of cultures on which single species and mixed species grew were calculated for bitches with positive bacterial tests on day 0 (n = 32) and day 63 (n = 36)

Bacterial growth was found in 84.2% of the samples on day 0 and in 94.7% of the samples on day 63. Mixed cultures were found in 63.1% of bitches with positive bacterial tests on day 0 and in 73.6% on day 63. Single species of bacteria were isolated in the cultures of 21.1% of samples from bitches with positive bacterial tests on both sampling days (Fig. 1).

A mean of 1.7 bacterial strains were isolated from the vaginas of bitches before the administration of the probiotic and a mean of 1.8 bacterial strains after the administration. Two species were found in 39.5% and 36.8% of the samples, three species in 21.1% and 26.3% of the samples and four species in 2.6% and 10.5% of the samples on day 0 and day 63, respectively (Table 1).

**Table 1.** Number of bacterial strains in the vaginas of Labrador retriever bitches (n = 38) before (day 0) and after (day 63) the oral administration of lactic-acid probiotic bacteria

Number of strains	Day 0		Day 63	
	number	%	number	%
0	6	15.8	2	5.3
1	8	21.1	8	21.1
2	15	39.5	14	36.8
3	8	21.1	10	26.3
4	1	2.6	4	10.5

**Fig. 2.**
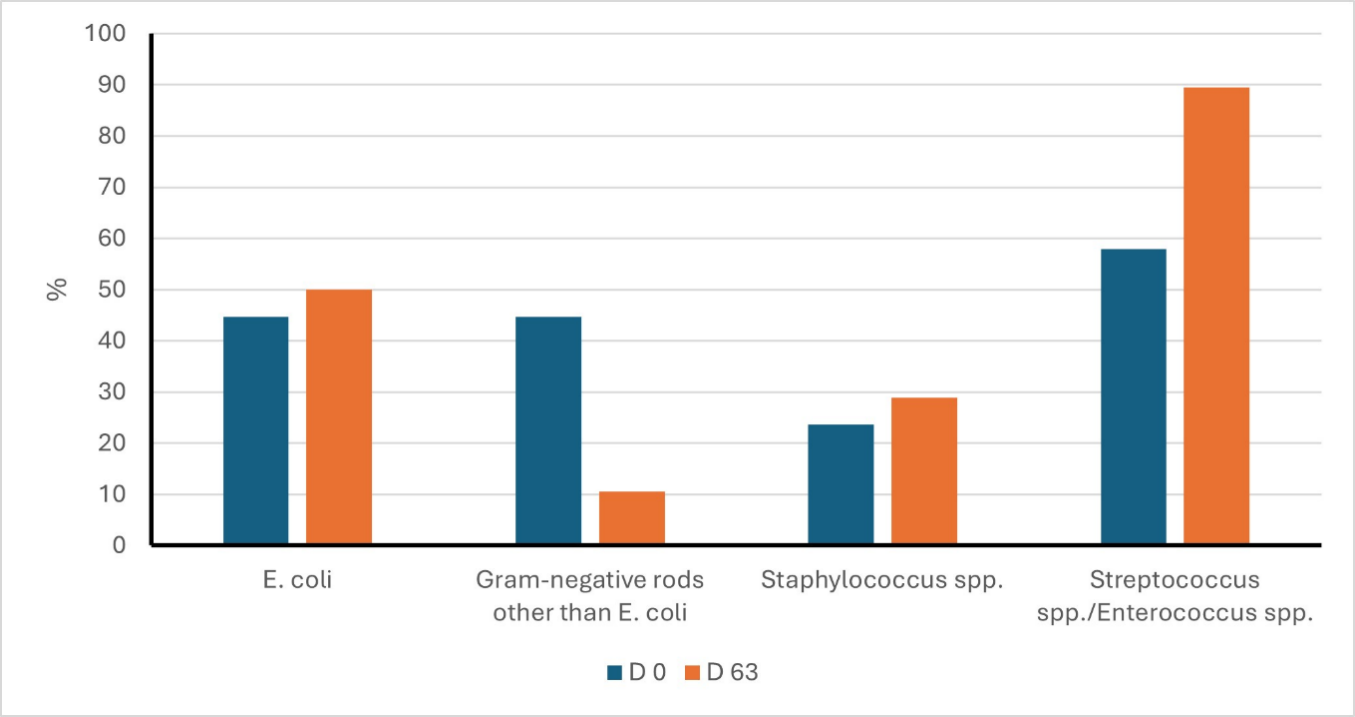
Vaginal bacterial populations of Labrador retriever bitches (n = 38) before (day 0) and after (day 63) the oral administration of lactic-acid probiotic bacteria. Values with different letters differ significantly at P-value < 0.05

There were some differences in the bacterial populations before and after the administration of the probiotic. *Escherichia coli* was isolated from the vaginal swabs of 44.7% and 50.0% of bitches, Gram-negative rods other than *E. coli* from 44.7% and 10.5% of swabs (P < 0.05), *Staphylococcus* spp. from 23.7% and 28.9% and *Streptococcus* spp./*Enterococcus* spp. from 57.9 and 89.5% on day 0 and day 63, respectively (P-value < 0.05) (Fig. 2).

Before the administration of the probiotic, *Clostridium perfringens* was found in 18.4% of bitches, *Canicola haemoglobinophilus* in 15.8% of them and *Pseudomonas aeruginosa* in 5.3%. These bacteria were not present in the vagina after the administration of the probiotic. The prevalence of *Streptococci* other than *Streptococcus canis* was significantly higher after the administration of the probiotic (26.3% *vs* 5.3%, P-value < 0.05). *Enterococcus* spp. were not found in the vaginas of bitches before the administration of the probiotic, whereas after the administration *Enterococcus* spp. were isolated from 28.9% of their samples (Table 2). Lactic-acid bacteria were not found before or after the administration of the probiotic. *Mycoplasma* spp. were not isolated because of the culturing techniques used.

**Table 2.** Bacteria isolated from vaginal swab samples of Labrador retriever bitches (n = 38) before (day 0) and after (day 63) the oral administration of lactic-acid probiotic bacteria

Bacteria	Day 0		Day 63	
	number	%	number	%
*Streptococcus canis*	21	55.3	23	60.5
*E. coli*	17	44.7^a^	19	50.0^b^
*Staphylococcus pseudintermedius*	9	23.7	11	28.9
*Enterococcus* spp.	-	-	11	28.9
*Clostridium perfringens*	7	18.4	-	-
*Canicola haemoglobinophilus*	6	15.8	-	-
*Proteus vulgaris*	2	5.3	4	10.5
*Pseudomonas aeruginosa*	2	5.3	-	-
*Streptococci* other than *Str. canis*	2	5.3^a^	10	26.3^b^

1 Values with different letters within a row differ significantly at P-value < 0.05

## Discussion

In this study bacteria were found in 84.2% of bitches before the administration of the probiotic and in 94.7% of bitches after the administration for two months. This is consistent with earlier studies revealing the presence of bacteria in the majority of vaginal samples from healthy bitches (4, 14, 24, 33, 41). This confirms that the vaginas of healthy bitches are colonised by physiological bacterial flora.

The mean number of bacterial strains was 1.7 before the administration of the probiotic and 1.8 afterwards. Cultures with single-species growth were obtained in the same proportion of swab samples taken from bitches before as after the administration of the probiotic (21.1% in both cases). This corresponds to the findings of Bjurström and Linde-Forsberg (4), who found a single species in the culture of 18.0% of healthy bitches. Lepes *et al*. (24) found the same in 43.7% of healthy and reproductive-diseased bitches. The clinical impact of single-species growth is still debated, as it is also often noted in cultures of samples from healthy bitches. However, Bjurström (3) and Jagódka *et al*. (20) cultured single species directly from inoculation more frequently from bitches with reproductive tract diseases than from healthy bitches. In our study the majority of such cultures consisted of *Streptococcus canis* (62.5% of pure cultures on day 0 and 100.0% on day 63). Bjurström and Linde-Forsberg (4) found *Pasteurella multocida* and beta-haemolytic *Streptococci* to be the most common isolates which grew without competitors in cultures of samples from breeding bitches. In the study of Groppetti *et al*. (14), the predominant bacteria isolated merely by inoculation in healthy bitches’ sample cultures were *Enterococcus faecalis* and β-haemolytic *Streptococcus* spp. *Escherichia coli* was the most frequent isolate colonising the medium alone in bitches with reproductive tract diseases (3, 16, 44).

In our study, mixed growths were found more frequently in cultures of samples from bitches after the administration of the probiotic (73.6% *vs* 63.1%). The mean number of bacterial strains isolated from the swabs of vaginas of bitches was similar (1.7 *vs* 1.8) at both sampling times. In healthy bitches, the number of isolates varied from 0.7 to 2.2 depending on sampling technique, site of sampling within the vagina and cycle stage (36).

In accordance with earlier studies (11, 14, 20, 24, 33) the most common bacteria found in the vaginas of bitches were *E. coli, Streptococcus canis* and *Staphylococcus pseudintermedius*. We found *Clostridium perfringens* in 18.4% of bitches before the administration of the probiotic. There are few studies on the prevalence of anaerobic bacteria in the vaginas of bitches. In the study of Watts *et al*. (41), anaerobic *Bacteroides* spp. were only isolated from individual bitches. In our study, *Lactobacillus* spp. were not isolated from the vagina before or after the administration of the probiotic. Generally, lactic-acid-producing bacteria of the *Lactobacillus* genus are not common isolates from the canine vagina (10, 19, 24, 27). This is associated with the high pH there, which ranged from 6.5 to 7.5 (7, 10).

The results of our study agree with those of Hutchinson *et al*. (19), which showed that the administration of the probiotic does not increase the prevalence of *Lactobacillus* spp. in the canine vagina. However, the oral administration of the probiotic for nine weeks resulted in some other changes in the composition of the vaginal flora. The prevalence of Gram-negative rods other than *E. coli* was significantly reduced. This is an interesting finding in the light of the report by Golińska *et al*. (11) of the presence of Gram-negative rods other than *E. coli* being significantly higher in bitches with genital tract infection than in healthy dogs. The potential pathogens *Clostridium perfringens, Canicola haemoglobinophilus* and *Pseudomonas aeruginosa* were eliminated, while the incidence of *Streptococci* other than *Streptococcus canis* was significantly increased. It is suggested that *Streptococcus* spp. protect the reproductive tract against pathogens by competing with them for nutrients and by interfering with adherence to epithelial cells (14). Therefore, these bacteria could play a defensive role against the pathogens most dangerous to canine fertility. *Enterococcus faecalis* was not found in the samples taken before the administration of the probiotic, but it was isolated from some after its administration. This was presumably due to changes in the intestinal flora of bitches caused by the probiotic, as demonstrated in the studies of Manninen *et al*. (29) and Gómez-Gallego *et al*. (12). Studies in women showed that there was a significant correlation between intestinal and vaginal bacterial flora (1, 8). In dogs, it has been shown that *E. coli* strains involved in pyometra displayed great similarity with *E. coli* isolates from the intestinal tract (16, 42). It is believed that there is translocation of intestinal bacteria to the vagina due to licking of the anogenital region (24).

*Mycoplasma* spp. were not isolated in this study because of the culturing techniques used, which was a limitation. The roles of these bacteria in inflammations of the reproductive tract and infertility are not fully established. According to several studies, *Mycoplasma* spp. are common inhabitants of the canine vaginal flora (20, 22, 28). On the other hand, they are isolated more frequently from bitches with genital diseases, or which have suffered abortion or stillbirth or produced weak puppies (34, 40). Many dog owners consider *Mycoplasma* spp. to be an obligatory pathogen and require a bacteriological test prior to mating, and if the bitch or stud dog is *Mycoplasma* spp. positive, they do not allow mating.

This study had other limitations: that the bitches belonged to different kennels and that different environmental factors affected them cannot be excluded as potential influences on the composition of their vaginal bacterial flora.

## Conclusion

The study showed that oral administration of lactic-acid probiotic bacteria for nine weeks did not increase the prevalence of *Lactobacillus* spp. in the vaginas of Labrador retriever bitches. There were changes in the composition of vaginal flora after the administration of the probiotic. The prevalence of Gram-negative rods other than *E. coli* was significantly reduced. The potential pathogens *Clostridium perfringens, Canicola haemoglobinophilus* and *Pseudomonas aeruginosa* were eliminated and the incidence of *Streptococci* other that *Streptococcus canis* and *Enterococcus faecalis* was significantly increased. Further research is needed to confirm the effect of the oral lactic-acid probiotic on the composition of the canine vaginal flora and its usefulness in the prevention of inflammatory diseases of the reproductive tract and infertility in bitches.
